# mRNA treatment produces sustained expression of enzymatically active human ADAMTS13 in mice

**DOI:** 10.1038/s41598-018-26298-4

**Published:** 2018-05-18

**Authors:** Susan Liu-Chen, Brendan Connolly, Lei Cheng, Romesh R. Subramanian, Zhaozhong Han

**Affiliations:** 1 Alexion Pharmaceuticals Inc., 100 College Street, New Haven, CT 06510 USA; 2 Alexion Pharmaceuticals Inc., 75 Sidney St, Cambridge, MA 02139 USA

## Abstract

Thrombotic thrombocytopenic purpura (TTP) is primarily caused by deficiency of ADAMTS13 within the blood stream due to either genetic defects or presence of inhibitory autoantibodies. Preclinical and clinical studies suggest that enzyme replacement therapy with recombinant human ADAMTS13 protein (rhADAMTS13) is effective and safe in treatment of TTP. However, frequent dosing would be required due to the relatively short half-life of rhADAMTS13 in circulation as well as the presence of inhibitory autoantibodies that collectively result in the poor pharmacological profile of rhADAMTS13. With technical breakthroughs in exploring mRNA as therapeutics, we hypothesized that restoration of ADAMTS13 activity for a prolonged duration of time can be achieved through systemic dosing of mRNA, wherein the dosed mRNA would utilize hepatic cells as bioreactors for continuous production of ADAMTS13. To test this hypothesis, mRNA encoding human ADAMTS13 WT or an ADAMTS13 variant, that had demonstrated resistance to predominant clinical TTP autoantibodies, was formulated in lipid nano-particles for liver-targeted delivery. In both ADAMTS13-sufficient and -deficient mice, a single dose of the formulated mRNAs at 1 mg/kg resulted in expression of hADAMTS13 at or above therapeutically relevant levels in mice for up to five days. This proof-of-concept study suggests that mRNA therapy could provide a novel approach for TTP treatment.

## Introduction

Thrombotic thrombocytopenic purpura (TTP) is a rare but life-threatening blood disorder characterized with clinical features such as thrombocytopenia, hemolytic anemia, fever, and multi-organ dysfunction. Without timely and appropriate treatment, patients with TTP have extremely high rates of morbidity or mortality^1^.

The underlying pathological mechanism that causes TTP has been identified as a severe deficit (<5% of normal enzymatic activity) of ADAMTS13 (a disintegrin and metalloprotease with thrombospondin type 1 repeats, member 13) within the blood stream^2,3^. This deficit occurs due to either genetic mutations within the ADAMTS13-encoding gene (inherited TTP, around 5% of the TTP population) or the presence of inhibitory autoantibodies (acquired TTP, around 95% of the TTP population). ADAMTS13 primarily drives cleavage of the polymeric ultra-large molecular weight von Willebrand factor (ULMW-vWF) into multimeric vWF of smaller sizes within blood vessels^4,5^. In patients with a severe ADAMTS13 deficit, overwhelming exposure of the thrombosis-prone ULMW-vWF on damaged endothelial cells leads to progressive aggregation of platelets and thrombus formation, which results in widespread microvascular ischemia also known as thrombotic microangiopathy or TMA, and subsequently all clinical symptoms of TTP^1,2,6^.

Currently available treatment options^1^ for inherited TTP include supplementing biologically active ADAMTS13 through plasma infusion. For acquired TTP, it is necessary to eliminate the inhibitory autoantibodies through plasma exchange with or without combination of B-cell depletion (e.g. Rituximab)^7^. Along with other experimental therapeutic options tested in preclinical models or clinical trials^8–14^, enzyme replacement with rhADAMTS13 has proved to be an effective and safe treatment for both inherited and acquired TTP^6,15^. In acquired TTP, there are high circulating levels of neutralizing antibodies to wild-type ADAMTS13 and physicians often must resort to immediate plasma exchange prior to addressing the underlying causality of TTP. However, frequent administration at high doses would be required due to the relatively short half-life of rhADAMTS13 in circulation^15–17^ and the presence of inhibitory autoantibodies^18,19^ that collectively result in a poor pharmacological profile of rhADAMTS13. Hence there remains a pressing need for better therapies for TTP patients, which could either decrease dosing frequency, or avoid microthrombi or completely abrogate the disease via gene therapy.

We hypothesized that providing mRNA-encoded ADAMTS13 would provide a steady supply of secreted, therapeutic protein for at least a few days and may be useful in treating patients even prior to a differential diagnosis of TTP or aHUS. In addition, glycosylation is known to be important for ADAMTS13 activity and we hypothesized that utilizing the hepatic/stellate cells to secrete ADAMTS13 may recapitulate their endogenous glycosylation profiles and have greater enzymatic activity than rhADMTS13. We report that human ADAMTS13 mRNA encapsulated in lipid nano-particles achieve extended duration of biologically active human ADAMTS13 protein in circulation. The mRNA was delivered into mouse liver and utilizes the hepatic cells as bioreactors for continuous production and secretion of ADAMTS13 into circulation. We also demonstrate that the ADAMTS13-M5 variant, which is not neutralized by autoantibodies, can be secreted and has greater enzymatic activity than ADAMTS13-WT in mice. This proof-of-concept study using an ADAMTS13 variant, that is resistant to inhibitory autoantibodies, suggests that mRNA represents a viable alternative approach for TTP treatment.

## Results

### Preparation and *in vitro* evaluation of mRNA

**Figure 1:**
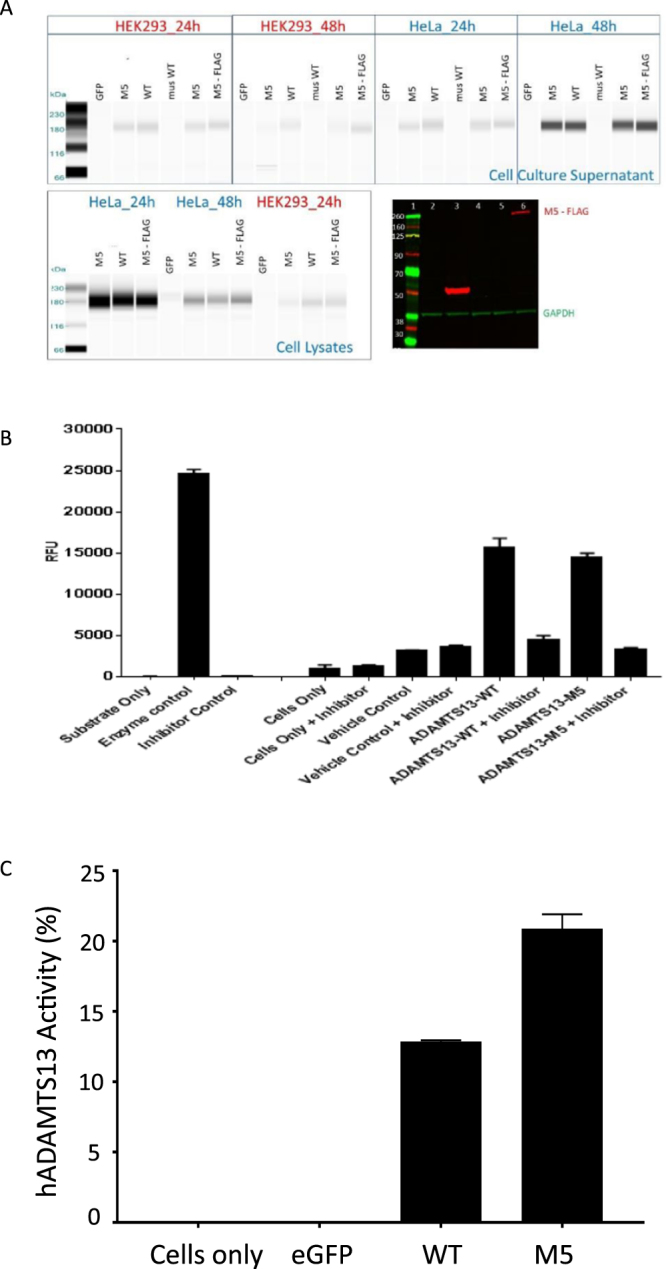
Characterization of mRNA-driven expression of rhADAMTS13. Shown are **(A**) capillary gel electrophoresis analysis of rhADAMTS13 protein expression (dark bands) in HeLa and HEK293 cell lysates and culture medium. rhADAMTS13 was detected with a rabbit monoclonal antibody against human ADAMTS13. Western blot analysis is shown for FLAG-tagged AdamTS13 M5 variant expression of HeLa cell lysates. GAPDH was detected with a specific mouse monoclonal antibody as a cell lysate loading control. Lanes 1–6 are respectively - Molecular weight standards, HeLa cells only, FLAG-tagged control protein (51 kDa), untagged M5, N-terminal FLAG-M5 and C-terminal FLAG-M5; (**B**) ELISA measurement of rhADAMTS13 in supernatant of transfected HeLa cells. rhADAMTS13 was quantified using native human ADAMTS13 as reference standard; (**C**) Enzymatic activity of rhADAMTS13 in supernatant of stellate cells after transfection with mRNA.

In this study, mRNA was synthesized using a proprietary optimized chemistry^20^ to encode the full length of human ADAMTS13 (hADAMTS13) with inclusion of its natural signal peptide for secretion. Analytical tests indicated that more than 98% of the mRNA transcripts had the theoretically calculated size of 4553 nucleotides, 100% of them had been capped at the 5′-terminus, the final mRNA products were endotoxin-free (<0.50 EU/mg) and negative in interferon-beta induction on fibroblast cells. Two constructs were generated to express the wild type (WT) or an autoantibody-resistant variant (M5)^21^ of human ADAMTS13. An eGFP-encoding mRNA prepared with the same protocol was used as a control. HeLa and HEK293 cells were selected for lipofection-mediated transfection to evaluate these mRNA. At 24- and 48-hours post-transfection, cell lysates and the filtered culture medium were collected and subjected to capillary gel electrophoresis analyses. It is observed that rhADAMTS13 with the expected molecular weight was detected in both the cell lysates and culture medium from the cells transfected with the ADAMTS13-encoding constructs, suggesting that the mRNA expressed and secreted rhADAMTS13 (Fig. 1A). The secreted rhADAMTS13 in the HeLa cell culture medium was further quantified with ELISA by using hADAMTS13-specific antibodies (Fig. 1B) and a fluorescence resonance energy transfer (FRET) assay by using a vWF segment as substrate (Fig. 1C). Such analyses indicated that both the WT and M5 variant of human ADAMTS13 had been expressed and secreted as enzymatically active forms. Similar results were also observed when these mRNAs were transfected into human primary hepatocytes (data not shown) or stellate cells **(**Fig. 1C**)**.

### *In vivo* administration of formulated rhADAMTS13 mRNA resulted in cellular uptake by hepatic cells as well as rhADAMTS13 expression and secretion into mouse circulation

**Figure 2:**
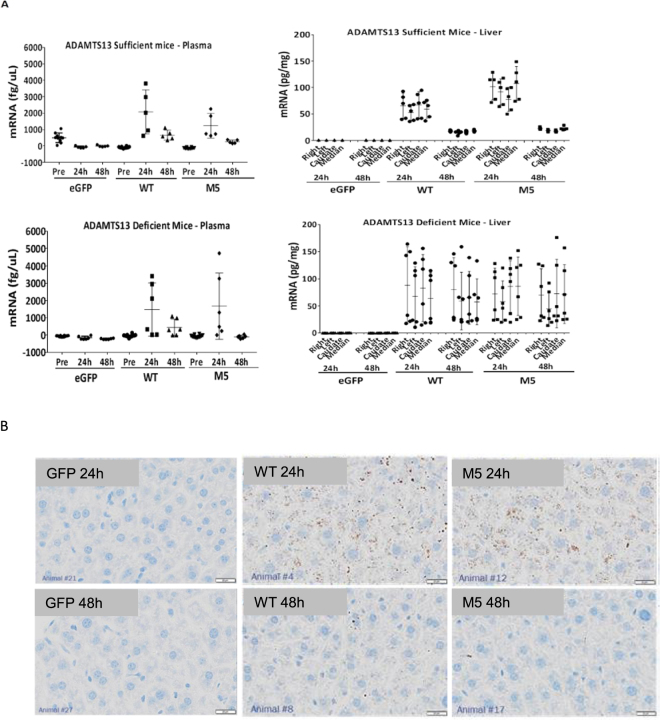
Distribution of hADAMTS13 mRNA within mouse plasma and liver. ADAMTS13-sufficient and –deficient mice were intravenously dosed at 1 mg/kg. Plasma and liver tissues were collected for hADAMTS13-specific mRNA quantitation at 24 and 48 hours post-injection. (**A**) qRT-PCR of hADAMTS13 mRNA in plasma and liver lysates; (**B**) *in situ* hybridization to show hADAMTS13-encoding mRNA (in brown) within liver sections. Representative images from multiple discontinuous slices from >2 liver lobes are presented, hematoxylin counterstaining (blue) shows cell nucleus.

To test the feasibility of using mRNA for *in vivo* production of biologically active proteins, the mRNA was formulated in lipid nano-particles with an average diameter of 95 nanometers^22^. Analytical tests indicated that more than 96% of the liposomal nanoparticles contained mRNA. The formulated mRNAs were intravenously administered in ADAMTS13-sufficient and -deficient mice at a dose of 1 mg/kg of body weight. Plasma and liver samples were collected at 24 and 48 hours post injection. Plasma samples were also collected from each animal before injection as baseline control. Quantitative measurements of the hADAMTS13-specific mRNA extracted from plasma and liver tissue lysates indicated that the mRNA had a concentration around 2 ng/mL (w/v) in plasma, and 100 ng/g (w/w) in liver at 24 hours post-injection. The hADAMTS13 mRNA was still detectable with concentrations close to baseline levels at 48 hours (Fig. 2A). There were no significant differences observed between the WT and M5 variant in the ADAMTS13-sufficient or –deficient mice regarding mRNA concentrations within plasma or liver.

The mRNA accumulation within mouse liver was further confirmed with *in-situ* hybridization (ISH) by using a panel of hybridization probes that had been optimized to specifically detect the mRNA encoding hADAMTS13. Analyses of multiple discontinuous slices from different liver lobes revealed a relatively even distribution pattern of the administered hADAMTS13 mRNA within mouse liver. Similar to the quantitative measurements with qRT-PCR, ISH studies also detected more of the human mRNA within hepatocytes at 24 hours post injection, while majority of the mRNA had been degraded by 48 hours (Fig. 2B).

**Figure 3:**
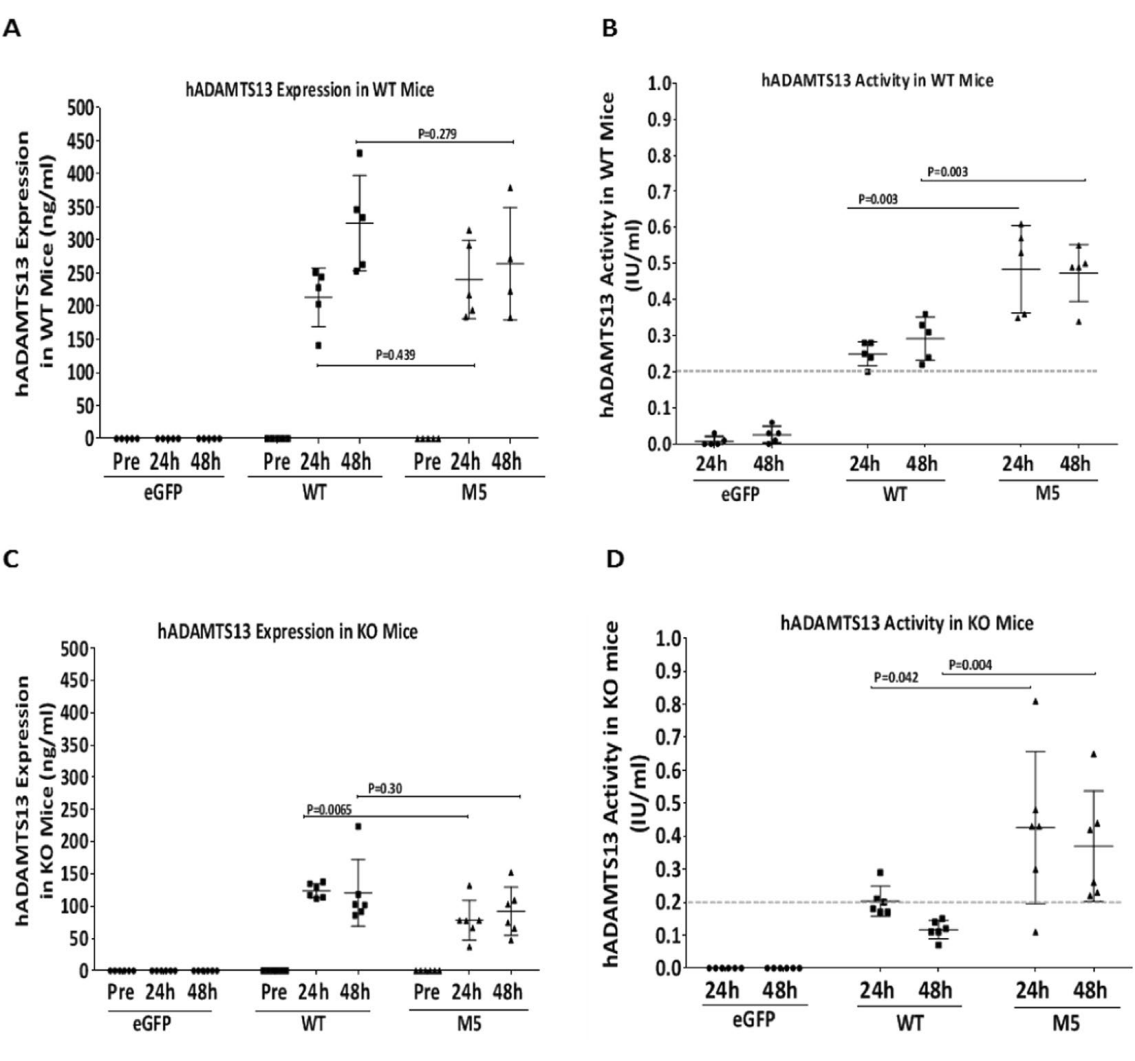
Quantitative measurement of rhADAMTS13 within mouse circulation after intravenous administration of mRNA. ADAMTS13-sufficient (**A**,**B**) and –deficient (**C**,**D**) mice were dosed at 1 mg/kg. Plasma samples were collected at 24 and 48 hours post mRNA administration for hADAMTS13 protein quantitation (**A**,**C**) and enzymatic activity level (**B**,**D**). *p < 0.05, n = 5 mice/group, Multiple *t* test.

Next, plasma samples were subjected to quantitative ELISA and enzymatic activity measurements to determine whether the mRNA within liver cells can drive expression of the encoded rhADAMTS13 and secretion into blood stream. In ADAMTS13-sufficient mice, ELISA measurements with hADAMTS13-specifc antibodies indicated that the WT as well as the M5 variant of hADAMTS13 protein had a concentration around 250 ng/mL in mouse plasma at 24 hours, and this hADAMTS13 concentration could be maintained at 48 hours post mRNA administration (Fig. 3A). In ADAMTS13-deficient mice, rhADAMTS13 protein had a concentration around 140 ng/mL in mouse circulation at 24 hours, and around 90 ng/mL at 48 hours post mRNA administration (Fig. 3B). Upon measuring enzymatic activity, we determined that more than 20% of the normal activity (standardized as 1 IU/mL) had been introduced and maintained for 48 hours in both the ADAMTS13-sufficient and –deficient mice after a single intravenous administration of hADAMTS13 mRNA at 1 mg/kg, particularly with the M5 variant of hADAMTS13. While comparing the WT and M5 variant of hADAMTS13, significantly higher enzymatic activity was observed with the M5 variant at similar protein levels, supporting the conclusion that the M5 variant possesses elevated enzymatic activity as demonstrated in the original report^21^.

### hADAMTS13 mRNA demonstrates prolonged duration of activity in mouse circulation

**Figure 4:**
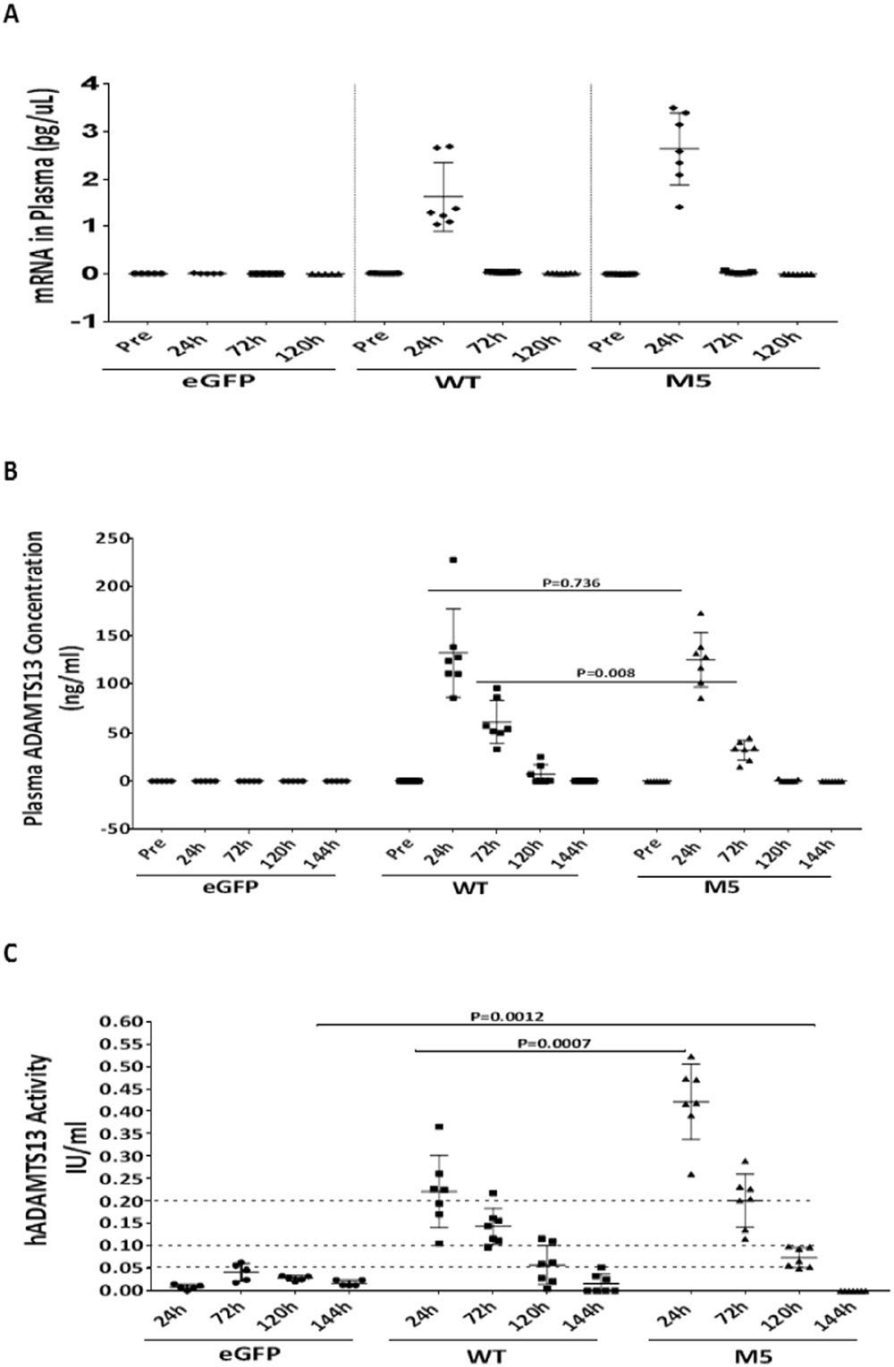
Extended single dose mRNA PK study and rhADAMTS13 quantification. ADAMTS13-sufficient mice were dosed at 1 mg/kg and plasma samples were collected at multiple time points as indicated. mRNA was quantified (**A**) with qRT-PCR by using hADAMTS13-specific probes, rhADAMTS13 protein was quantified with ELISA (**B**) by using hADAMTS13-specific antibodies, and the enzymatic activity (**C**) of rhADAMTS13 were measured as described. *p < 0.05, n = 7 mice/group, Multiple *t* test.

A single dose pharmacokinetic study of the liposome-encapsulated mRNA in ADAMTS13-sufficient mice was conducted with inclusion of time points up to 120 hours post intravenous administration at 1 mg/kg. qRT-PCR measurements indicated that the formulated mRNA had a plasma concentration of 1–4 ng/mL at 24 hours, but was barely detectable at 72 hours post injection (Fig. 4A). However, a remarkable amount of rhADAMTS13 protein, either the WT or the M5 variant, was still detectable in mouse plasma at these time points (Fig. 4B). More interestingly, the enzymatic activity of rhADAMTS13 was still significant (>5% of the normal levels) at 120 hours after the administration of mRNA (Fig. 4C). A rough calculation indicated that the rhADAMTS13 activity, either the WT or M5 variant, decayed 50% every 48 hours. Since rhADAMTS13 has a terminal half-life in mouse circulation of around 24 hours^16^, we concluded that systemically dosed mRNA had utilized liver cells as bioreactors for continuous production of rhADAMTS13 and thus established and maintained rhADAMTS13 activity for an extended period of time.

## Discussion

TTP is a rare but life-threatening disease with high morbidity and mortality rates in the absence of timely and appropriate treatment. The current standard of care is plasma exchange, however multiple therapeutic approaches such as anti-inflammatory antibodies, proteasome modulators, antioxidants, anti-VWF nanobodies and gene therapy have been explored^8–14^. Among these experimental therapeutic options, enzyme replacement therapy (ERT) has shown promise in preclinical models and *ex vivo* studies^6,15^. However, frequent dosing would be needed due to the relatively short half-life of rhADAMTS13 in mice (24 hours)^16^ or humans (2–3 days)^17^ and due to the presence of inhibitory autoantibodies against hADAMTS13^18,19^. By using mRNA encoding the wild type or an autoantibody-resistant M5 variant of human ADAMTS13 we demonstrate that longer duration of action is possible *in vivo*. Our study suggests that ERT of ADAMTS13 with infrequent or episodic dosing, e.g. upon infection driven flares of TTP, would be technically feasible with an mRNA-based therapeutic approach.

It has been reported that around 95% of the TTP patients have ADAMTS13-inhibitory autoantibodies in circulation^18,19,23^. Existence of such inhibitory autoantibodies would create another hurdle in delivering a meaningful ADAMTS13 enzyme replacement therapy, as the autoantibodies could either directly prevent ADAMTS13 from enzymatically cleaving its substrate ULMW-vWF, or indirectly reduce its bioavailability through antibody-antigen complex formation and altered pharmacokinetics. To address these challenges, multiple approaches have been proposed, including developing ADAMTS13 variants that resist autoantibody binding^21,24^, encapsulation within platelets for targeted drug release^25^, or anchoring ADAMTS13 to the damaged endothelial cells^26^ that represent the pathological sites of TTP. Among those approaches, the M5 variant of hADAMTS13 stands out to be attractive for TTP treatment. Based on structural and functional information of hADAMTS13 protein and its interaction with inhibitory autoantibodies, five point mutations were rationally designed and introduced to eliminate the epitopes recognized by the inhibitory autoantibodies. *Ex vivo* studies with clinical samples indicated that the M5 variant of hADAMTS13 was resistant to inhibition by more than 80% of the autoantibodies from patients who had been diagnosed as ‘acquired TTP with significant amounts of hADAMTS13-inhibiting autoantibodies’. Interestingly, the M5 variant demonstrated superior enzymatic activity when compared to native human ADAMTS13^21^, which would allow enhanced therapeutic efficacy with less amount of recombinant protein in circulation. In our study, elevated enzymatic activity was also observed in the M5 variant of hADAMTS13 at equivalent protein levels as those of the wild type hADAMTS13. Further studies are warranted to assess any immunogenicity associated with the M5 variant protein in primates or humans.

Our studies indicate that rhADAMTS13 can be produced and secreted by HeLa, HEK293 and stellate cells (Fig. 1). Importantly, secreted ADAMTS13 protein is the appropriate molecular weight and retains enzyme activity (Fig. 1A,B) which lends confidence for evaluation *in vivo*. Our data also indicate that ADAMTS13 is expressed at high levels in HeLa cells as seen in the 24 h cell lysate data (Fig. 1A). Furthermore, secretion of ADAMTS13 is delayed with the highest levels seen in culture medium at 48 h post-transfection (Fig. 1A). We also demonstrate that the C-terminal FLAG tag is detectable and not deleterious for ADAMTS13 expression & secretion, as evidenced by the lack of expression of the N-terminal FLAG-M5 construct (Fig. 1A). We hypothesize that the N-terminal FLAG tag may interfere with appropriate glycosylation, folding or transport of ADAMTS13 protein. *In vivo* studies in ADAMTS13-sufficient mice (WT) indicates that hADAMTS13 mRNA can be detected in plasma as well as liver at 24 h with a return to just above baseline levels at 48 h (Fig. 2A,B). An interesting finding is that in ADAMTS13-deficient mice, the hADAMTS13 mRNA appears to be present in the liver at relatively high levels even at 48 h (Fig. 2A – lower right panel). This raises the possibility that a negative feedback loop exists in ADAMTS13 expressing cells/tissues whereby exogenous or additional recombinant ADAMTS13 may be ineffective or downregulated. This observation needs further studies to assess if such a feedback loop exists in primates or humans. In our study we detect prolonged ADAMTS13 enzymatic activity up to 5 days and this could be a result of the prolonged duration of the M5 variant in mouse liver, thus allowing sustained ADAMTS13 protein production and secretion of active protein into circulation (Fig. 3A–D). Subsequent single dose duration of action studies in WT mice recapitulated the presence of WT and M5 hADAMTS13 mRNA and protein in plasma (Fig. 4A,B), however the M5 variant demonstrated higher enzyme activity over time (Fig. 4C). This result corroborates data that suggests the M5 variant can produce greater hADAMTS13 enzyme activity per ng of protein. We speculate that in acquired TTP, wherein significant levels of autoantibodies to ADAMTS13 are present, dosing of the M5 variant mRNA may provide sustained levels of enzyme activity to decrease ULMW-vWF and prevent thrombotic microangiopathies. Taken together, in our study we demonstrate that mRNA-encoded ADAMTS13 can be synthesized, delivered to hepatic cells via LNPs and translated into functional protein *in vivo*.

Our study also suggests that more efficient intracellular delivery of the mRNA would be needed. In this study, around 20 µg of the formulated mRNA was injected intravenously at a dose of 1 mg/kg, which would translate to ~10 µg/mL in circulation with an assumption of blood volume of 2 mL. At 24 hours post injection, only around 2 ng/mL, or 0.02% of the dosed rhADAMTS13 mRNA was detected in mouse circulation. At that time point, roughly 100 pg/mg of the hADAMTS13 mRNA was detected in mouse liver tissue lysate, totally 100 ng or 0.5% of the dosed mRNA within mouse liver with an assumption of liver weight of 1 gram. While our data suggests that the formulation enabled liver-specific accumulation of hADAMTS13 mRNA, we also speculate that improvement of mRNA chemistry^27^, formulation^28^, delivery route^29,30^, mRNA translation efficiency^31^ and protein longevity^32^ would be required to improve mRNA bioavailability and thus an enhanced pharmacological profile of the expressed protein. mRNA can provide a rapid delivery and translation of effective protein, however translation efficiency and duration of protein activity needs to be improved. Current research suggests that altering UTRs can increase efficiency of mRNA translation and produce greater amounts of active protein^31^. In parallel, protein engineering to increase half-life of the protein may supply adequate, effective protein over a long duration of time so as to enable infrequent dosing^32^.

In the past few years, mRNA has emerged as an alternative approach to protein-based therapeutics. mRNA is amenable to large scale production and is relatively stable when compared to the corresponding recombinant protein. The mRNA-based approach utilizes host cells as bioreactors for protein production thereby ensuring appropriate post-translational modification (e.g. glycosylation) and also minimizes some risks associated with product heterogeneity introduced in manufacturing and processing of recombinant proteins. As illustrated in this study, utilizing host cells as a bioreactor for continuous production of the mRNA-encoded protein would be extremely beneficial for therapeutic proteins that have a relatively short half-life in circulation. In fact, utilization of mRNA as an alternative therapeutic approach has been successfully explored in multiple reports, including anti-viral vaccination^33^, vascular regeneration after myocardial infarction^20^, monoclonal antibody treatment of HIV-1 infection^34^, and factor IX replacement for treatment of hemophilia B^22^. Our study provides a novel avenue whereby mRNA can be applied for *in vivo* production of a secreted protein as large as hADAMTS13, which is composed of 1427 amino acids, and potentially provide another treatment option for patients suffering from Thrombotic thrombocytopenic purpura.

Taken together, our data indicates that mRNA-encoded ADAMTS13 provides active protein *in vitro* and *in vivo*, and this protein was active for 5 days *in vivo*. These are encouraging results considering rhADAMTS13 generally lasts for a day in mice and 2–3 days in humans. Further work to increase protein expression and duration of action is required, but we can postulate that the ADAMTS13-M5 variant may be able to last for longer than 5 days in humans and provide significant clinical benefit to acquired TTP patients.

## Materials and Methods

### mRNA synthesis and formulation

Human ADAMTS13 (hADAMTS13) mRNA was synthesized as described^20^ using a proprietary optimized chemistry to encode the full length of hADAMTS13 with inclusion of its natural signal peptide for secretion. Two constructs of hADAMTS13 were generated to express the wild type (WT) or an autoantibody-resistant M5 variant (M5)^21^. An eGFP-encoding mRNA was synthesized with the same procedures as the control. All mRNAs were formulated into lipid nano-particles (LNP) with an average diameter of 95 nanometers.

### Cell culture and transfection

HeLa and HEK293 cells were obtained from ATCC (Manassas, VA); and human hepatic stellate cells from Creative Bioarray (Shirley, NY). Monolayer cell culture was prepared per supplier instructions for mRNA transfection. Briefly, cells with 80% confluency were transfected with 2.5 µg of each mRNA and 4 uL Lipofectamine™ MessengerMAX™ transfection reagent (Thermo Fisher Scientific, Inc., Waltham, MA). Cell lysates and culture media were collected at the indicated time points for capillary gel electrophoresis or SDS-PAGE followed by immunoblotting with anti-hADAMTS13 (rabbit mAb ab177940, 1:2,000) or anti-GAPDH (mouse mAb ab125247, 1:6,000, both from Abcam, Cambridge, MA) and detection with the corresponding secondary antibodies. The collected culture media were also subjected to quantitative measurements of rhADAMTS13 as below.

### Quantitative measurement of hADAMTS13

hADAMTS13 protein concentrations were measured with Quantikine ELISA Kit (R&D Systems, Minneapolis, MN) by following manufacturer’s protocol. Enzymatic activities of hAdamts13 within culture media or mouse plasma were measured with SensoLyte® 520 ADAMTS13 Activity Assay Kit (Anaspec Fremont, CA) or TECHNOZYM® ADAMTS-13 Activity ELISA (Diapharma West Chester, OH), respectively, by following the procedures provided by the manufacturers.

### *In vivo* studies

All experiments, animal housing, handling and experimental procedures/protocols were approved and performed in accordance with Alexion Pharmaceuticals Inc. IACUC guidelines and regulations. ADAMTS13-sufficient and -deficient mice with B6129SF1/J genetic background were obtained from The Jackson Laboratory and maintained in a pathogen–free facility. 10–16 week old mice with mixed gender were used to evaluate single dose mRNA pharmacokinetics, expression and activity of hADAMTS13. Each animal received intravenous injection of the formulated mRNA at 1 mg/kg, in a total volume of 200 uL as freshly prepared solution in PBS. Before and after the injection, submandibular bleeding was used to collect blood samples at the indicated time points, while terminal bleeding was conducted by cardiac puncture. Whole blood was collected into microtubes with 3.2% tri-sodium citrate solution at 9:1 ratio; plasma samples were prepared by centrifugation at 1500 rcf for 5 minutes at 4 °C and stored at −70 °C before quantitative measurements of mRNA and hADAMTS13. To collect liver tissues, animals were fasted for 4 hours before necropsy. After cardiac perfusion with saline, all the liver lobes were collected and one 5 mm punch from each lobe was snap-frozen in liquid nitrogen and stored at −70 °C for mRNA extraction and quantification. Median liver lobe was fixed in 10% formalin for 48 hours and then transferred to PBS to have tissues sections for *in-situ* hybridization. All experiments, animal housing, handling, dosing, sample collection and experimental procedures were performed in accordance with Alexion Pharmaceuticals Inc. IACUC guidelines and regulations.

### Plasma and liver mRNA quantification

mRNA from mouse plasma or liver tissue lysates were quantitatively measured with branched DNA (bDNA) assay using QuantiGene Singleplex kit (Affymetrix, Santa Clara, CA). A panel of detection probes were designed against the target mRNA sequence, assay was validated as instructed by the manufacturer using purified mRNA as reference standard prior to quantitating the mRNA within mouse plasma or liver tissue lysates.

### *In situ* hybridization (ISH)

ISH was conducted on Leica Bond Rx (Leica) by using ACD ISH LS2.5 DAB (Advanced Cell Diagnostics Inc., Newark, CA). Briefly, slides were baked at 60 °C for 60 min, and dewaxed and rehydrated according to Leica protocol. To expose mRNA properly while maintaining tissue morphology, target sequence retrieval was performed at 95 °C for 15 min followed by proteinase K treatment at 40 °C for 15 min. Probe hybridization was performed at 40 °C for 120 min. Signals were visualized with DAB substrate after multiple signal amplification steps, followed by counterstaining with hematoxylin. Slides were then taken off the machine and serially rinsed with 70%, 80%, 90% and 100% ethanol (Fisher Scientific). After cleaning in xylene (Fisher Scientific), slides were mounted with Cytoseal (Fisher Scientific) and observed under bright field (Nikon).

### Statistics

Multiple *t* test was applied for statistical analyses for all the quantitative data, with parameter setting as unpaired, not assuming consistent SD, and correcting for multiple comparisons using the Holm Sidak method. *P* values smaller than 0.05 or 0.01 were considered to have statistically significant or very significant differences between the groups, respectively. All graphs were prepared with GraphPad Prism version 7.00 (GraphPad Software Inc., La Jolla, CA).
